# Evaluation of district mental healthcare plans: the PRIME consortium methodology

**DOI:** 10.1192/bjp.bp.114.153858

**Published:** 2016-01

**Authors:** Mary J. De Silva, Sujit D. Rathod, Charlotte Hanlon, Erica Breuer, Dan Chisholm, Abebaw Fekadu, Mark Jordans, Fred Kigozi, Inge Petersen, Rahul Shidhaye, Girmay Medhin, Joshua Ssebunnya, Martin Prince, Graham Thornicroft, Mark Tomlinson, Crick Lund, Vikram Patel

**Affiliations:** **Mary J. De Silva**, MSc, PhD, **Sujit D. Rathod**, MSC, PhD, Centre for Global Mental Health, London School of Hygiene and Tropical Medicine, London, UK; **Charlotte Hanlon**, MRCPsych, PhD, Department of Psychiatry, College of Health Sciences, Addis Ababa University, Addis Ababa, Ethiopia and Centre for Global Mental Health, Health Services and Population Research Department, Institute of Psychiatry, Psychology and Neuroscience, King's College London, London, UK; **Erica Breuer**, MPH, Alan J Flisher Centre for Public Mental Health, Department of Psychiatry and Mental Health, University of Cape Town, Cape Town, South Africa; **Dan Chisholm**, PhD, Department of Mental Health and Substance Abuse, World Health Organization, Geneva, Switzerland; **Abebaw Fekadu**, MD, PhD, MRCPsych, Department of Psychiatry, College of Health Sciences, Addis Ababa University, Addis Ababa, Ethiopia and Department of Psychological Medicine, Centre for Affective Disorders and the Affective Disorders Research Group, Institute of Psychiatry, Psychology and Neuroscience, King's College London, London, UK; **Mark Jordans**, PhD, Centre for Global Mental Health, Health Services and Population Research Department, Institute of Psychiatry, Psychology and Neuroscience, King's College London, London, UK and HealthNet TPO, Research and Development Department, Amsterdam, The Netherlands; **Fred Kigozi**, MBChB, MMed, Butabika National Referral and Teaching Hospital/Makerere University, Kampala Uganda; **Inge Petersen**, PhD, School of Applied Human Sciences, Howard College, University of KwaZulu-Natal, South Africa; **Rahul Shidhaye**, MD(Psych), MHS, Centre for Mental Health, the Public Health Foundation of India, India and Maastricht University/CAPHRI School for Public Health and Primary Care, Maastricht, The Netherlands; **Girmay Medhin**, MSc, PhD, Department of Psychological Medicine, Centre for Affective Disorders and the Affective Disorders Research Group, Institute of Psychiatry, Psychology and Neuroscience, King's College London, London, UK and Aklilu Lemma Institute of Pathobiology, Addis Ababa University, Addis Ababa, Ethiopia; **Joshua Ssebunnya**, MSc, Butabika National Referral and Teaching Hospital/Makerere University, Kampala, Uganda; **Martin Prince**, MD, MSc, MRCPsych, Centre for Global Mental Health, Health Services and Population Research Department, Institute of Psychiatry, Psychology and Neuroscience, King's College London, London, UK; **Graham Thornicroft**, FRCPsych, PhD, Centre for Global Mental Health, Health Services and Population Research Department, Institute of Psychiatry, Psychology and Neuroscience, King's College London, London, UK; **Mark Tomlinson**, BA, BA(Hons), MA(ClinPsych), PhD, Centre for Public Mental Health, Department of Psychology, Stellenbosch University and Department of Psychiatry and Mental Health, University of Cape Town, Stellenbosch, South Africa; **Crick Lund**, BA, BSocSci(Hons), MA, MSoSci(ClinPsych), PhD, Alan J Flisher Centre for Public Mental Health, Department of Psychiatry and Mental Health, University of Cape Town, Cape Town, South Africa, and Centre for Global Mental Health, Institute of Psychiatry, Psychology and Neuroscience, King's College London, UK; **Vikram Patel**, PhD, MRCPsych, FMedSci, Centre for Global Mental Health, London School of Hygiene and Tropical Medicine, London, UK, Centre for Mental Health, the Public Health Foundation of India, India and Sangath, Goa, India

## Abstract

**Background**

Few studies have evaluated the implementation and impact of real-world mental health programmes delivered at scale in low-resource settings.

**Aims**

To describe the cross-country research methods used to evaluate district-level mental healthcare plans (MHCPs) in Ethiopia, India, Nepal, South Africa and Uganda.

**Method**

Multidisciplinary methods conducted at community, health facility and district levels, embedded within a theory of change.

**Results**

The following designs are employed to evaluate the MHCPs: (a) repeat community-based cross-sectional surveys to measure change in population-level contact coverage; (b) repeat facility-based surveys to assess change in detection of disorders; (c) disorder-specific cohorts to assess the effect on patient outcomes; and (d) multilevel case studies to evaluate the process of implementation.

**Conclusions**

To evaluate whether and how a health-system-level intervention is effective, multidisciplinary research methods are required at different population levels. Although challenging, such methods may be replicated across diverse settings.

The real-world evaluation of mental health programmes is essential to understand how and why programmes work – or do not work, and which are the most promising for scale up.^1^ However, very few high-quality evaluations have been carried out in low- and middle-income countries (LMIC),^2^ where it can be argued the need for scaling up services is greatest.^3^ A recent systematic review of evaluations of scaled-up mental health programmes found 136 evaluations globally, only 15 of which were conducted in LMIC.^4^ Many of these did not evaluate key domains such as effect on patient outcomes, the process of implementation or cost outcomes of the programme.

The aim of the PRogramme for Improving Mental health carE (PRIME) is to generate research evidence on the implementation of district-level mental healthcare plans (MHCPs) for five target mental, neurological and substance use disorders comprising depression, maternal depression, alcohol use disorders, psychosis and epilepsy in Ethiopia, India, Nepal, South Africa and Uganda.^5^ This paper describes the multidisciplinary cross-country research methods that the PRIME consortium is employing to evaluate the coverage, impact and process of implementation of the MHCPs. The goal of PRIME is to use these evaluations to generate evidence that can be widely adopted by policy makers and practitioners, and is the first project to comprehensively evaluate mental health programmes delivered at scale in a range of low-resource settings.

## Method

The research methods used in PRIME were developed over a period of 3 years by the consortium including all country teams and cross-country partners. From the proposal development stage, PRIME has been a partnership between academic researchers and Ministries of Health in the five countries, along with cross-country research partners. Representatives of the Ministries of Health attended the meetings where the methods were developed to ensure that the results are relevant for local policy making. They also play a key role facilitating the implementation of the MHCPs. Group consensus was achieved on the key cross-county components of the evaluation, with working groups established to develop methods in detail. Country teams then piloted and further refined the methods before finalisation. Flexibility was built into the cross-country protocols, allowing countries to add research questions of particular policy relevance in that setting.

### Evaluation framework

The MHCPs implemented by the five countries are complex service delivery and health system interventions at the community, primary healthcare facility and district health organisation level.^6–10^ A comprehensive evaluation is therefore necessary to answer the question ‘what works for whom in what context?’ The PRIME evaluation takes place at four levels: (a) population (contact coverage); (b) facility (impact on detection of disorders); (c) patient (effect on patient outcomes); and (d) district (the process of implementation within the broader social, political, economic and cultural context).

**Fig. 1:**
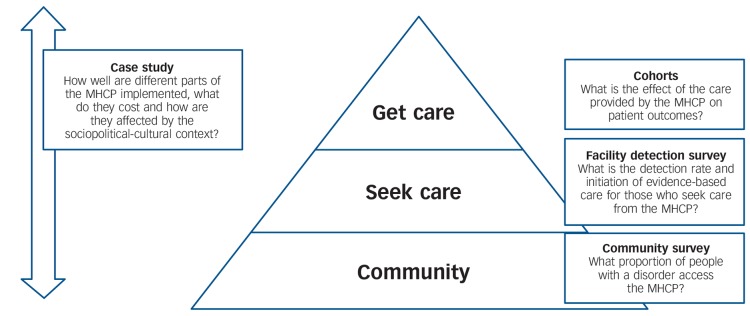
Overview of PRogramme for Improving Mental health carE (PRIME) study designs. MHCP, mental healthcare plan.

To achieve this we developed a comprehensive set of evaluation methodologies nested within a conceptual framework to explore cross-country research questions. The UK Medical Research Council's framework for the evaluation of complex interventions^11^ combined with the theory of change (ToC)^12^ was used to generate a comprehensive cross-country evaluation of all stages of the MHCPs. The Medical Research Council's framework describes four iterative stages of development, feasibility/piloting, evaluation, and implementation of complex interventions.^11^ A ToC is visually represented in a causal pathways map that describes exactly how an intervention is expected to achieve its impact within a particular setting and facilitates the identification of specific points of the intervention pathway that can be evaluated.^13,14^ Within PRIME, a cross-country ToC was developed to describe the causal pathways through which the integration of mental health services into primary healthcare is expected to lead to improved patient outcomes.^15^ The use of ToC is described in detail in Breuer *et al* in this supplement.^16^ Briefly, a series of steps (preconditions) that need to be achieved in order to improve patient outcomes are displayed as a causal pathway (for example primary healthcare workers have the required skills to be able to correctly diagnose mental, neurological and substance use disorders). The outcomes are converted into measurable indicators (for example proportion of people correctly diagnosed with depression). These indicators are then turned into research questions with research methods designed to test these, covering all of the indicators on the ToC. In this way, a comprehensive evaluation of all stages of the MHCPs is generated, which can be used across countries. Figure 1 and Tables 3 and 4 in Breuer *et al* in this supplement detail the PRIME ToC, indicators for each outcome on the ToC and the study design used to measure each indicator (see also online Fig. DS1).^16^

### Research questions

The broad cross-country research questions generated from the PRIME ToC are as follows.

- What is the impact of the programme on access to healthcare?
- What is the level of detection and initiation of evidence-based care in those who access care?
- What are the clinical, social and economic outcomes for those who are identified as having a target mental, neurological and substance use disorder?
- How well is each component of the MHCP delivered?

## Results

A suite of four studies was developed to answer the key cross-country research questions, capturing all the cross-country indicators from the cross-country ToC. These comprise: (a) community survey: a repeat cross-sectional community survey to measure contact coverage for adults with depression or alcohol use disorders; (b) facility detection survey: repeat cross-sectional facility-based survey to measure detection of depression or alcohol use disorders and initiation of correct treatment; (c) treatment cohorts: follow-up of patients diagnosed with depression, alcohol use disorders, psychosis or epilepsy to assess the change in a range of individual patient outcomes, with additional follow-up of caregivers of people with psychosis or epilepsy; and (d) case study: a range of qualitative and quantitative methods to evaluate the process of implementing the MHCPs in each district. Figure 1 illustrates how these four studies combine to measure contact coverage, detection, patient outcomes and implementation processes.

### Cross-country evaluation methods

Table 1 lists the key cross-country research questions and principal methods for each study design. A number of strategies are being implemented to ensure fidelity of methods across countries.

**Table 1:** Summary of PRogramme for Improving Mental health carE (PRIME) research questions and methods

Research questions	Cross-country methods
Among adults who screen positive for depression or alcohol use disorders in the community, estimate the: change in equitable coverage of treatment for any current episode of depression or alcohol use disorders Among the general population in the community, estimate: change in the prevalence of depression and alcohol use disorders change in level of stigma, discrimination and mental health knowledge change in patterns of help-seeking for mental, neurological and substance use disorders compared with other health conditions change in healthcare and time costs associated with help-seeking for adults with depression/alcohol use disorders *v*. other health conditions	Repeat population-based cross-sectional survey of adults in the district Community survey of between 1500 and 2040 adults in each country conducted at baseline before the mental healthcare plans (MHCPs) have been implemented and approximately 36 months later.

Among adults attending primary healthcare facilities, estimate the: prevalence of depression/alcohol use disorders among primary healthcare attendees level of detection (correct diagnosis) of depression/alcohol use disorders initiation of appropriate evidence-based treatment for adults diagnosed with depression/alcohol use disorders equity of detection and correct initiation of treatment by severity of disability, gender and socioeconomic status	Repeat cross-sectional survey of primary healthcare attendees Conducted: (a) before the MHCPs are implemented; (b) repeated at least once after the MHCP has been implemented; and (c) at the end of the implementation phase Between 760 and 1900 adults screened in each country

Among adults with priority conditions treated by the MHCP, estimate the: change in severity of clinical symptoms change in economic outcomes change in social functioning change in experience of stigma and discrimination equity of treatment provision by gender and socioeconomic status describe the clinical/social services received and the pathways through care of patients treatment uptake, retention in care and adherence to treatments satisfaction with healthcare services	Separate 12 month cohorts for each disorder 200 adults with depression treated by the MHCP, 200 with alcohol use disorders, 150 with psychosis and 150 with epilepsy interviewed immediately following diagnosis and again at 3 or 6 months and 12 months in each country. Cohort of caregivers for people with psychosis and epilepsy Qualitative interviews with adults in the cohort and their caregivers

In the districts in which the MHCPs are being implemented, evaluate: the feasibility, acceptability and costs of the PRIME MHCPs the health system requirements for scaling up - human resources, training, supervision needs, infrastructure, drugs and budget whether the MHCPs were implemented as intended how the physical, social, political or environmental contexts in which the MHCPs were implemented affected or interacted with the implementation of the PRIME MHCPs	Range of qualitative and quantitative methodologies Annual profiles of the districts, facilities and communities where the MHCPs are being implemented including resource costing of MHCP activities mplementation logs collated monthly Evaluation of the quality of training and supervision of primary healthcare workers Qualitative interviews with healthcare providers and patients to assess the process of implementation

First, a core set of data-collection tools are used across all study designs to ensure that key domains such as patient outcomes are measured consistently to facilitate comparisons across countries between the different study designs and across time. These data collection tools are outlined in Table 2. Efforts were made to choose tools that had been validated in the PRIME countries and, where these did not exist, if possible to conduct validation studies and back-translation to ensure they were suitable for use in the PRIME districts (B. Kohrt, personal communication, 2015; and references^17–30^). Although the same screening tools are used across countries to facilitate comparison, country or regional-specific validated cut-offs are used to ensure that the tool is measuring a valid construct in that country. Country research teams are led by a principal investigator, with day-to-day management of the research team carried out by a country-coordinator with extensive experience of managing research projects in-country. Data-collection teams comprise fieldwork coordinators (normally with a Masters degree), supervisors and fieldworkers, the majority of whom have a Bachelors degree. All data-collection staff receive specific training to understand the research protocol and administration of the PRIME instruments.

**Table 2:** Outcome assessment tools used across the PRogramme for Improving Mental health carE (PRIME) studies

Domain	Instrument
Clinical symptoms	
Depression	Patient Health Questionnaire 9 (PHQ-9)^20,21^
Alcohol use disorders	Alcohol Use Disorders Identification Test (AUDIT)^22^
Psychosis	Brief Psychiatric Rating Scale (BPRS)^23^
Epilepsy	Two-question instrument developed by PRIME: How long ago did you last experience a seizure? (days, weeks, months, years ago) How many seizures did you have in the past 30 days?

Social and economic outcomes	
Disability	World Health Organization Disability Assessment Schedule 2.0 12-item (WHODAS 2)^24^
Economic status	Employment status, income, ability to do work and household chores from WHODAS 2 36-item^24^
Knowledge, attitudes and behaviour	Mental Health Knowledge Schedule (MAKS)^25^
	Reported Intended Behaviour Scale (RIBS)^26^
	Community Attitudes toward Mental Illness (CAMI)^27^
Healthcare expenditure	Client Service Receipt Inventory (CSRI)^28^
Discrimination	Discrimination and Stigma Scale (DISC-12)^29^
Internalised stigma	Internalised Stigma of Mental Illness (ISMI) subscales^30^

Second, the same inclusion criteria for participants in the studies are being used across countries. These comprise being above the age of majority in the country; the ability to communicate in a local study language; and the willingness and ability to provide informed consent (or permission from the caregiver in the case of people with psychosis who are too unwell to provide informed consent).

Finally, we standardised data collection and data management as much as possible through using mobile telephones for data collection in all countries apart from Ethiopia where paper questionnaires with double data entry into Epidata version 3.1.0^31^ was considered more feasible because of the logistical challenges of using mobile telephones in a rural district with erratic internet connectivity and power supply.

The four study designs are outlined in more detail below. Country-specific variations are not presented in this paper as the focus is on the cross-country research methods. The full protocols are available on the PRIME website (www.prime.uct.ac.za).

#### Community survey

The community survey is a repeat cross-sectional study designed to measure the change in population-level contact coverage (the proportion of people who have a disorder who receive treatment for that disorder) of services for depression and alcohol use disorders before and 3 years after implementation of the MHCPs. As psychosis and epilepsy are uncommon, it is not possible to estimate contact coverage with adequate precision using the community survey as we would need to screen large numbers of people to detect enough cases. Instead, contact coverage for psychosis and epilepsy are calculated from health management information system (HMIS) records on the number of people diagnosed and treated for epilepsy in the PRIME clinics, divided by estimates of the prevalence of the disorders in the population.

All countries apart from South Africa are conducting the community survey. In South Africa, integration of mental health into integrated chronic disease management was prioritised by the South African Department of Health.^9^ As the overwhelming majority of people with communicable and non-communicable diseases using the chronic care service access care on a regular basis from primary healthcare facilities,^32^ these facilities, rather than the community, provide an appropriate location for assessing treatment coverage of mental, neurological and substance use disorders in these individuals.

For both depression and alcohol use disorders, the inputs to the sample size calculation are the estimated (pre-MHCP) baseline contact coverage, the (post-MHCP) expected contact coverage and the prevalence of the disorder, to detect the expected change in contact coverage with 80% power and two-sided alpha of 0.05. As screening tools are used to assess who may need services, the sample sizes are adjusted to account for the possibility of false positives diluting the real change in treatment coverage effect, by incorporating site-specific figures for the positive predictive value of each screening tool. Population-based sampling strategies were used to select a random sample of adults in the district where the MHCPs are implemented. In Ethiopia, a district-level census list enabled simple random sampling of households and adults within that household. In the other countries where no census was available, recent estimates of village population sizes were used to randomly select villages. Within those villages, households and individuals within these households were randomly selected. The sample sizes for each country are: Ethiopia (*n* = 1500), India (*n* = 1855), Nepal (*n* = 2040) and Uganda (*n* = 1800). A paper presenting full details of the methods and results of the community survey is in preparation (S. Rathod, personal communication, 2015).

In the survey, a screening questionnaire is administered to every participant to provide basic sociodemographic information and to screen for depression and alcohol use disorders. Respondents are not asked whether they have depression or alcohol use disorders specifically as these terms are rarely used in the settings. Instead, respondents are administered a screening tool (Patient Health Questionnaire (PHQ-9) for depression^20,21^ and Alcohol Use Disorders Identification Test (AUDIT) for alcohol use disorders^22^), which are checklists of symptoms of these disorders. If they screen positive, they are asked about whether they sought help for these problems recently, rather than giving these clusters of symptoms a diagnosis. Approximately 90% of the participants who screen negative on both screening tools finish the interview at this stage. In Uganda and Nepal, all participants who screen positive on either screening tool plus a random sample of 10% of those who screen negative complete the full interview. In India and Ethiopia, all respondents irrespective of case-status complete the full interview. This comprises sections on detailed sociodemographics; mental health-related knowledge, attitudes and behaviour; disability; treatment sought and received in the past 12 months for the mental health problem; and general healthcare use. This enables us to determine whether contact specifically for mental disorders is improved by the MHCPs.

#### Facility detection survey

The facility detection survey is a repeat cross-sectional study designed to measure the sensitivity and specificity of primary healthcare workers' diagnosis of depression and alcohol use disorders. Depending on the country, a baseline detection survey is conducted prior to implementation of the MHCP, approximately 6 months after the primary healthcare worker training is complete, and 2 years after the baseline survey. All eligible adult clinic attendees from clinics in the implementation district were approached and those that consented to participate were recruited. The sample size calculation inputs for the facility detection survey are broadly similar to the community survey.

As with the community survey, interviewers administer a short screening interview to a sample of adults attending clinics. The sample sizes for each country are: Ethiopia (*n* = 1014), India (*n* = 760), Nepal (*n* = 1467), South Africa (*n* = 1243) and Uganda (*n* = 1900). The sample sizes vary between countries because the prevalence of depression and alcohol use disorders in the clinics varies, as does the expectation of the impact that the MHCP will have on detection rates. Respondents who screen positive for harmful alcohol use disorders and/or current depression or depression in the past 12 months will complete a section on help sought in response to recent symptoms of the respective disorder, and any reasons for a delay in help-seeking. All participants who screen positive on either screening tool, plus a random sample of 10% of those who screen negative have data on diagnosis and treatment extracted from clinical records or a non-directive purpose-built form filled in by the clinician to ascertain: (a) whether the primary healthcare worker made a diagnosis of depression and/or alcohol use disorders; and (b) whether the primary healthcare worker initiated an appropriate treatment plan or provided an appropriate referral to specialist care. To validate the measurement of whether a diagnosis was given and treatment initiated, all countries apart from Ethiopia are also collecting information from the patient through a post-consultation interview that asks the participant whether they received a diagnosis, treatment plan or referral.

Transparent country-specific criteria for defining diagnosis and provision of an appropriate treatment plan are being used. For determining whether the correct diagnosis was given these comprise: was the term depression, alcohol use disorders or the contextually equivalent term used? If no medical term was used, was the clinician's description of the problem consistent with such a diagnosis? For determining whether an appropriate treatment plan was provided, one of the following criteria must be met: (a) relevant advice for reducing symptom severity; (b) an appropriate specialist referral; (c) an appropriate medication regimen; or (d) a combination of (a), (b) or (c), as determined by locally recognised clinical standards of care.

#### Treatment cohorts

The treatment cohorts are designed to assess the clinical, social and economic outcomes of patients with a target mental, neurological and substance use disorder treated by the MHCP. Up to five cohorts, comprising adults diagnosed with depression, alcohol use disorders, psychosis or epilepsy are followed up in each implementation area to estimate changes in clinical, social and economic functioning over 12 months. Table 3 lists the key methods used in the cohort studies.

**Table 3:** Overview of cohort methods

	Depression	Alcohol use disorder	Psychosis	Epilepsy
Countries conductin cohort	Ethiopia, India, Nepal, South Africa, Uganda	Ethiopia, India, Nepal	Ethiopia, India, Nepal (caregivers only),	Ethiopia, Uganda, Nepal

Target population	Adults who have been newly diagnosed with depression or alcohol use disorders by a primary healthcare provider in the PRIME implementation areas		Adults who have a new or existing diagnosis of psychosis or epilepsy by a primary healthcare provider in the PRIME implementation areas	

Sampling	Consecutive or systematic sampling of diagnosed patients from primary care clinics in the PRIME implementation areas			
			Each patient will be asked to identify their primary adult carer who will be asked to participate in a caregiver cohort	

Inclusion criteria	New diagnosis of depression b a primary healthcare provider in the implementation area	y New diagnosis of alcohol use disorders by a primary healthcare provider in the implementation area	New or existing diagnosis of psychosis by a primary healthcare provider in the implementation area	New or existing diagnosis of epilepsy by a primary healthcare provider in the implementation area
	Ability to speak local study language			
	Time and ability to complete the full interviews			
	Residency in the PRIME implementation area for 12 months following recruitment			
	Adults with ability to provide informed consent: age above age of majority in the country (i.e. 16 years or 18 years; and willingness to provide informed consent)			
			Those without ability to provide informed consent (epilepsy or psychosis): willingness to provide assent and informed consent from an adult caregiver on their behalf	

Data collection methods	Interviewer-administered questionnaire at baseline, mid-point and end-point			
			Interviewer-administered questionnaire to caregiver at baseline, mid-point and end-point Clinical interview at baseline, mid-point and end-point (optional)	

Mid-point visit	3 months after baseline (+/−2 weeks)		6 months after baseline (+/−2 weeks)	3 months after baseline (+/−2 weeks)

End-point visit		12 months after baseline (+/-4 weeks)		

Sample size	≥200	≥200	≥150 patients,+ ≥150 caregivers	≥150 patients,+ ≥150 caregivers

Primary outcomes	Symptom severity (Patient Health Questionnaire 9)	Symptom severity (Alcohol Use Disorders Identification Test with 3-month recall) Problems related to drinking	Symptom severity (Brief Psychiatric Rating Scale)	Symptom severity: number of seizures in past 30 days Number of days since last seizure
	Disability (World Health Organization Disability Assessment Schedule II 12-item)			

PRIME, PRogramme for Improving Mental health carE.

All adults with a target disorder and living in the implementation area are eligible for inclusion. People with depression and/or alcohol use disorders will be recruited from primary healthcare facilities when they are first diagnosed, whereas people with epilepsy or psychosis will be recruited from a range of sources including community case-finding using locally developed methods, audits of patients already receiving services, and those who are newly diagnosed in facilities implementing the MHCP. In addition, although only newly diagnosed cases of depression and alcohol use disorders will be included, people with an existing diagnosis of epilepsy or psychosis are eligible for inclusion. This is because it is probable that some existing cases are already known to services in districts where there are existing mental health services and these individuals will therefore be referred to the newly implemented MHCP services. In addition, the primary caregiver (defined as the adult who is primarily responsible for helping the patient to meet daily needs) of people with epilepsy and psychosis will be asked to participate in cohorts of caregivers to assess any changes in caregiver burden or economic status after treatment. The recruitment window will close on accumulation of the required sample size for the cohort.

Without a control group we cannot state that the change in clinical scores is attributable to the MHCP. Thus, the cohort sample sizes are set to detect a meaningful within-person reduction in symptom severity with 80% power and two-sided alpha of 0.05. Sample size calculations show that we can detect a meaningful clinical impact for treatment of depression and alcohol use disorders with 200 individuals in each cohort, and for treatment of psychosis and epilepsy with 150 individuals in each cohort. Not all countries are implementing all cohorts, with the choice of disorders to evaluate dependent on the disorders targeted by the MHCPs^33^ and the feasibility of collecting the required sample size for the cohort. For example, Uganda and South Africa are not doing an alcohol use disorders cohort as results from the facility detection survey indicate that the number of people presenting with alcohol use disorders in primary care is too low to recruit 200 people over the available period of time.

High retention of cohort participants is essential to evaluate the impact of the MHCP. Participants who have refused treatment, discontinued clinical care, and/or are not adherent to treatment will remain in the cohort; these participants are of particular interest as they provide critical information with regard to gaps in the MHCP. The country research teams will actively contact participants for follow-up, using means that have been agreed on as part of the informed consent process (for example, telephoning, home visits and contact through a third party). The mid-point outcome assessment is the time point at which the optimal effect of treatment is expected to occur. For depression and alcohol use disorders, the optimal effect is expected 3 months and for psychosis and epilepsy 6 months after baseline. The end-point is 12 months for all cohorts, to capture social and economic outcomes by accounting for seasonality and the likelihood that improvements in social and economic functioning will follow improvements in clinical status.^34^

A wide range of information from interviews with patients and caregivers at repeated time points enables a detailed analysis of the baseline moderators and mediators that affect programme effectiveness for individual patients (for example, severity of symptoms, socioeconomic status, gender, which interventions were received and how adherent patients were to them). Key outcomes for the analysis include clinical, social and economic outcomes; equity of access, stigma and discrimination; adherence and retention in care; referral pathways; and patient satisfaction.

#### Case studies

As the broader social, political, economic and cultural context may affect the implementation and therefore the impact of the MHCPs, we are also assessing these contextual factors. A variety of methods will be used for a case study of each district MHCP. Methods used include document reviews, individual in-depth interviews with patients, caregivers and service providers, studies of supervision quality, facility profiles, training fidelity, costing studies and context monitoring.

The case studies are designed to measure a specific set of input, process and output indicators as identified by the PRIME ToC,^16^ which are not otherwise captured by the community survey, facility detection survey and the cohorts. Specifically, the case studies measure the availability of physical, human and financial resources required for the implementation and monitoring of the MHCPs; the implementation of the MHCPs, including the supervision and training of people implementing the MHCPs; and the context in which the MHCPs operate. Four different methods drawing on multidisciplinary methods are being used to collect these data, with results combined across studies. These comprise the following.

- Profiles of the districts, facilities and communities in which the PRIME MHCPs are being implemented. The full profiles will be collected annually, with selected indicators collected quarterly including the information needed to cost the MHCPs.
- PRIME implementation logs: tally sheets of the training conducted and interventions that are implemented, collected on a monthly basis.
- Evaluation of PRIME training and supervision: completed concurrently with the training of primary healthcare workers and linked to the facility detection survey.
- PRIME process evaluation: in-depth interviews with service providers to evaluate the acceptability, feasibility and process of implementation of the MHCPs. The exact composition will depend on the country, but will include the main groups of healthcare providers involved in the MHCP such as community-based workers, primary healthcare workers and mental health specialists. Sampling will be purposive across professional categories, and for rural and urban health facilities, aiming for at least 6–8 from each category per country, or until data saturation is reached. Topics covered include: lessons learned from the experience of implementing the MHCP to improve delivery, the supervision system and the acceptability of delivering mental healthcare in primary care.

In addition, over 400 in-depth interviews with patients and caregivers including those who do not engage in treatment will evaluate the acceptability and feasibility of the MHCPs. Purposive sampling is used based on disorder, level of engagement in care and type of treatment received, with oversampling of vulnerable groups (particularly women and those living in poverty). The final sample size will be determined by when data saturation is achieved. Topics covered include: the acceptability of the care provided; barriers to adhering to treatment; experience of stigma and discrimination; and the perceived relationships between treatment and functional and economic status. All interviews will be conducted either at the facility where the patient is receiving care or in their home. Interviews with patients and service providers are timed for 6 months after the start of the cohort studies to allow time for stabilisation of mental state and also for all participants' to have had experience of delivering or receiving care.

### Overall evaluation of the MHCPs

The four designs outlined above provide evidence as to the performance of particular aspects of the MHCPs (for example, contact coverage or detection). The use of ToC allows us to combine the results from these different studies into one conceptual framework to comprehensively evaluate the entire MHCP. Results will be mapped on to the PRIME ToC to determine how well the MHCPs were implemented in each context, and the impact that this had on the effectiveness of the MHCP. This will enable us to revise the country-level ToCs, and ultimately the cross-country ToC, to reflect how the MHCPs were actually implemented and the pathways that led to patient outcomes.

In addition, results from different study designs can be combined to answer major research questions. This includes the cost of the MHCP per healthy-life-year-gained for each disorder, calculated by combining data on the cost of the MHCP from the case studies with disability outcomes from the cohorts. In addition, the effective coverage of the MHCPs (the proportion of people who require an intervention who derive benefit from it)^4^ will be estimated by combining coverage from the community survey with outcomes from the cohorts. If this information is combined with data from the facility survey that indicates that, for example, women with depression are being systematically underdiagnosed, and results from the case studies that show that primary healthcare staff did not sufficiently retain their skills post-training, we can begin to understand how the impact of the MHCPs can be attributed to the process of implementing the MHCPs.

## Discussion

The PRIME evaluation is rigorous and comprehensive, involving four study designs to assess contact coverage, detection and the effect on patient outcomes as well as a full process evaluation to understand what works for whom and in what context. The four study designs combine to give us a picture of the overall functioning of the MHCP. Importantly, we are pioneering the use of ToC as a conceptual framework to provide a cohesive structure to the evaluation, enabling a set of cross-country research questions and associated methods to be developed, with flexibility for countries to answer locally relevant questions or apply different methods. We are using this structure to apply multidisciplinary and multilevel methods in order to understand how context affects implementation and, ultimately, patient outcomes in different settings.

### Challenges faced implementing the evaluation

There are a number of challenges that we have faced in developing such a complex, multilevel and multidisciplinary evaluation. Critical among these is the challenge inherent in developing methods that are suitable across a range of country settings and that are potentially replicable elsewhere. In particular, the use of a common approach for screening for mental, neurological and substance use disorders across five countries may be problematic. In addition to questions related to the validity of using the same measurement tool across cultures and settings, country teams face considerable issues in translating questions from instruments to be culturally relevant, and training lay fieldworkers to appropriately administer these tools. Contextual challenges also mean that not all aspects of the evaluation design can be applied uniformly across the different country contexts. For example, in Uganda and South Africa, the low prevalence of alcohol use disorders in people attending primary healthcare clinics resulted in an alcohol use disorders cohort not being included in the cohort design of these countries.

Second, we face a number of logistical challenges related to conducting research in low-resource settings, including developing a community sampling plan where no census data are available, the lack of valid and reliable mental, neurological and substance use indicators in HMISs, the risk of overburdening participants and staff at the health facilities, and building research capacity in each site to conduct the evaluation.

Third, the Hawthorne effect,^35^ whereby research activity positively affects the effectiveness of the intervention, is a significant issue, particularly in the facility detection survey. Knowing that the content of patients sessions is being assessed may directly affect the behaviour of clinicians, leading them to improve their diagnostic skills through a heightened awareness of mental health. This bias has been countered by keeping research methods as separate as possible from the MHCP interventions. For example, individual patient scores on the screening tools are not reported to the clinicans unless the patient is suicidal, so the only way that primary healthcare staff can improve their diagnositic skills is through the training and supervision structures set up as part of the MHCP.

Finally, PRIME is restricted to before–after evaluations in cohort studies to measure the effect on patient outcomes, instead of randomised controlled trials. This is because of limited research funds and ethical considerations (such as limited ‘usual care’ options for a control arm when there is effectively no existing care in many settings).

Although the baseline measurement of indicators will be compared with the results after implementation, there is no control group of people with diagnosed disorders who do not receive the MHCP to take into account other changes within the health system or wider context that may influence patient outcome. We have attempted to counter this limitation in two ways. First, we have developed a PRIME ToC that specifies the mechanism by which we expect the MHCPs to achieve their impact. We are measuring the indicators on the theoretical causal pathways between the programme interventions and the observed impact, thus enabling mediation analyses for the outcomes; it will therefore be possible to attribute changes in the outcomes to the strength of the intervention implementation. To aid attribution of effect to the MHCPs, Nepal and Uganda are also following up a comparison cohort of patients who screen positive for depression or alcohol use disorder using the PHQ-9/AUDIT, but who are not identified and treated by the MHCP and are therefore not enrolled in the treatment cohorts. Second, we are exploring the social, political and environmental context in which the MHCPs are being implemented. This will facilitate a greater understanding of the other changes occurring in the district that could be contributing to the observed impact and allow us to more accurately attribute the impact observed to the MHCPs. Non-randomised studies such as PRIME are acceptable when randomisation is not possible for logistical or ethical reasons, when a similar intervention does not already exist in the population of interest and when the size of the effect is expected to be large.^36^ As no or very few mental health services were available in the districts before the MHCPs were implemented, it is likely that any changes to patient outcomes will be because of the MHCPs rather than external factors.

### Future plans

The methods PRIME is employing are comprehensive and intensive, and are only feasible as a research evaluation as opposed to a routine evaluation of a mental health programme. It is clear that a balance must be struck between the effort needed to implement a programme and the effort needed to evaluate it. After this implementation phase, when the MHCPs are scaled up in the last phase of PRIME, less intensive evaluation methods will be developed relying more heavily on strengthened HMISs to collect routine data on service utilisation and changes in patient outcomes, with nested studies to explore the process of implementation and scale up.

PRIME is applying the same methods to five very different country contexts to compare and contrast the integration of mental health into primary care in low-resource settings. The methods we have developed are intended to be used by other researchers who wish to evaluate real-world mental health programmes in other settings.
